# Negotiating power relations, gender equality, and collective agency: are village health committees transformative social spaces in northern India?

**DOI:** 10.1186/s12939-017-0580-4

**Published:** 2017-09-15

**Authors:** Kerry Scott, Asha S. George, Steven A. Harvey, Shinjini Mondal, Gupteswar Patel, Kabir Sheikh

**Affiliations:** 1 Public Health Foundation of India, Delhi, 122022 India; 2 Johns Hopkins School of Public Health, Baltimore, 21205 USA; 3 University of the Western Cape, Cape Town, 7535 South Africa; 4 The University of Newcastle, Newcastle, NSW 2308 Australia

**Keywords:** Village health committee, Participation, Agency, Gender, Power, Social spaces, Discourse, India

## Abstract

**Background:**

Participatory health initiatives ideally support progressive social change and stronger collective agency for marginalized groups. However, this empowering potential is often limited by inequalities within communities and between communities and outside actors (i.e. government officials, policymakers). We examined how the participatory initiative of Village Health, Sanitation, and Nutrition Committees (VHSNCs) can enable and hinder the renegotiation of power in rural north India.

**Methods:**

Over 18 months, we conducted 74 interviews and 18 focus groups with VHSNC members (including female community health workers and local government officials), non-VHSNC community members, NGO staff, and higher-level functionaries. We observed 54 VHSNC-related events (such as trainings and meetings). Initial thematic network analysis supported further examination of power relations, gendered “social spaces,” and the “discourses of responsibility” that affected collective agency.

**Results:**

VHSNCs supported some re-negotiation of intra-community inequalities, for example by enabling some women to speak in front of men and perform assertive public roles. However, the extent to which these new gender dynamics transformed relations beyond the VHSNC was limited. Furthermore, inequalities between the community and outside stakeholders were re-entrenched through a “discourse of responsibility”: The comparatively powerful outside stakeholders emphasized community responsibility for improving health without acknowledging or correcting barriers to effective VHSNC action. In response, some community members blamed peers for not taking up this responsibility, reinforcing a negative collective identity where participation was futile because no one would work for the greater good. Others resisted this discourse, arguing that the VHSNC alone was not responsible for taking action: Government must also intervene. This counter-narrative also positioned VHSNC participation as futile.

**Conclusions:**

Interventions to strengthen participation in health systems can engender social transformation. However they must consider how changing power relations can be sustained outside participatory spaces, and how discourse frames the rationale for community participation.

## Background

Health committees are a commonly promoted mechanism for community participation in health [1] and there is some evidence that they can improve the functionality and accountability of health facilities [2–5] and increase community use of health services [6, 7]. In addition to directly improving health systems and health-related behavior, participation through health committees is envisioned to play a broader emancipatory role for communities and is seen as a valuable end in itself [8–10]. However, health committee functionality and effectiveness is very uneven [11–13], with many committees failing to achieve inclusive and sustained community engagement [7, 14, 15].

Researchers suggest that many of the disappointing outcomes of participatory development programs can be explained by failure to manage power inequalities, both within communities and between communities and outside stakeholders such as policymakers [8, 16, 17]. Within communities, power inequalities can prevent marginalized groups from benefiting from the participatory programs seeking to help them [18–22]. Some participatory programs have particularly failed to manage gender inequalities, excluding women from decision making while still expecting their participation in interventions they did not design [23]. Power inequalities between communities and outsiders have been identified as another major issue, with elites (i.e. government policymakers, officials, program implementers) accused of using community participation initiatives to push external agendas, overburden communities with unreasonable responsibilities, or legitimize failures in public service provision [24–28].

This paper explores how power inequalities play out through village health, sanitation, and nutrition committees (VHSNCs) in rural north India in order to understand the transformative potential of these social spaces to support new, more equitable, power relations and enable collective local action for improved health. VHSNCs were initiated as part of India’s National Rural Health Mission in 2005, with a goal of forming one committee per village. The policy encourages participation by community members in health promotion activities and strengthens community linkages with government health, sanitation, and nutrition services. With extensive global interest in health committees [1, 29] and over 500,000 VHSNCs formed across India [30], it is vital to understand the ways in which health committees influence power relations (such as around gender) within communities and how the rationale for participation framed by outside stakeholders influences community collective agency.

## Methods

### Conceptual framework

This paper is framed by the concept of “social spaces” [31] to examine how power works within health committees. Social spaces are interactive moments in space and time, constructed through relationships between diverse groups, which create contexts for new social representations and identities to emerge [32, 33]. Thinking about the health committee as a social space through which power relations mediate interactions between diverse actors provides a fruitful lens into how participatory programs can support communities to construct more health-enabling social identities [34].

**Fig. 1:**
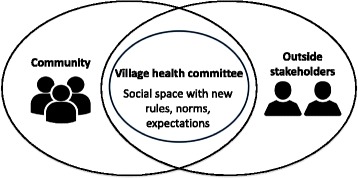
Village health committees as social spaces created through interaction among diverse stakeholders

Producing a new space, such as a health committee, can create a momentary disruption of established rules and possibilities, into which unfamiliar rules and alternative possibilities can be ascribed [35]. VHSNCs gather people who do not normally meet (i.e. men and women, people from different castes and religions, local leaders and young mothers) within a set of rules and procedures that people do not typically follow (i.e. specific topics to discuss, roles for members). What happens within this alternative social space affects the broader society when participants experience incongruence between the norms within the space (such as ‘all voices are equal’) and the norms governing interactions in regular life (‘male voices are more valued’), leading them to question and challenge established ways of being and interacting [36] (Fig. 1).

Inherent to the concept of social spaces is a post-modern understanding of power, where power is inseparable from the development of accepted knowledge systems and that power/knowledge is articulated through discursive norms [22, 35, 37]. Discursive norms are the socially accepted communicative tools that shape and limit what is said and done, what is say-able and do-able, and ultimately what is considered truth or knowledge [37, 38]. For example, elite actors may control the parameters of village health committee discussions and actions by influencing the discursive norms governing which topics are discussed and how.

Yet power is complex and unstable; every site where power is expressed contains the potential for resistance [37]. Discourse is thus not only “an instrument and an effect” of power, but is also “a stumbling point of resistance and a starting point for an opposing strategy” [38]. Despite attempts by more powerful actors to control participatory social spaces, such as village health committees, marginalized people can still deploy their own resistance tactics to further their “alternative visions” about how best to “transform their possibilities” [22]. For example, Mosse [39] explores the ways in which communities may outwardly agree to stakeholder agendas, but then exercise “quiet power” [40] to ensure programs suit their own needs.

### Setting

VHSNCs bring together a range of village residents (Table 1). Membership should include 50% women and adequate representation of people from marginalized social groups, particularly scheduled castes (SCs) and scheduled tribes (STs).

**Table 1:** Description of VHSNC members

VHSNC member	Description
General community members	• Includes members of savings groups and school management groups
Auxiliary nurse midwife (ANMs)	• Frontline health worker, female, works from health sub-center covering five or more villages (member of VHSNC in her own village, and invited guest for the VHSNCs in the other villages she serves)
Accredited social health activist (ASHA)	• Community health worker, female, one per village (1000 population)
Anganwadi staff	• Preschool and nutritional supplementation workers, female, two per village anganwadi (preschool)
Ward member	• Lowest elected member of local government (*panchayat*)

The VHSNC is to convene monthly meetings, develop health plans, take action to improve environmental health, monitor and seek improvements in public services, and allocate a yearly “untied fund” of Rs. 10,000 (US $150). The untied fund is a grant for community health action, revolving emergency loans, or to help destitute families with health care.

Since their launch in 2005, VHSNC functionality has remained low [41, 42]. Members often did not know their roles, VHSNC composition rarely adhered to guidelines, meetings were often irregular or not occurring, and there was often negligible community participation in budgeting, monitoring, or developing health plans [41, 43, 44].

In 2013, the Indian Ministry of Health and Family Welfare (MoHFW) developed a support package to bolster VHSNC activity [30]. This package involved: village social mobilization to increase community knowledge of VHSNCs, expanding VHSNC membership from 7 to 15 people, training members, and facilitating monthly village meetings and quarterly cluster meetings. Before scaling up the support package nationally, in-depth implementation research at the block (i.e. sub-district) level in northern India was undertaken. The findings reported here emerged from this implementation research study.

The study took place in “Manujpur”,^1^ a rural block of approximately 300,000 people within 250 km of New Delhi [45]. Most people work as farmers and laborers. Residents struggle to access sufficient water and experience many shortcomings in public services. Roads often become impassible in the rainy season, there is little public transportation, public schools are considered to be of low academic and physical quality, and government health centers are severely understaffed. The literacy rate is 80% for men and 50% for women [45].

The patriarchal system in the region limits women’s decision-making, access to education, and freedom of movement. Most women avoid allowing senior male in-laws to hear them speak or look at their faces (called *purdah*) by remaining silent in the presence of male community members, covering their faces with cloth (called *ghungat*), and lowering their heads or turning towards a wall whenever a senior man was nearby. Women sit on lower surfaces than men, generally squatting on the ground when men sit on chairs, and women eat after men have finished. These practices were not performed with outsiders, such as the male researcher and male NGO staff (who were from nearby villages); women sat on chairs across from them, and could speak directly to them without covering their faces.

The local government (*panchayat*) reserves one-third of all elected seats for women. However, men and women openly report that whenever an area has to elect a woman, the names of female candidates on the ballot are proxies for male relatives, who then perform all functions in place of the elected woman.

While gender is an obvious shaper of identity, family wealth, caste, and religion also affect identity performance. These aspects of identity are expressed in innumerable ways, including: the people with whom you could speak, which parts of the village you could enter, whose foods you could eat, and the community roles for which you were eligible. For instance, most higher caste people do not eat food or drink tea served by lower caste people and when a group is seated together on the ground, higher caste people often sit on the rug while lower caste people sit on the bare floor.

### Data collection

Over the course of 1.5 years (2014–2015), a local non-governmental organization (NGO) called "SEEK" implemented the VHSNC support package in 50 of the 200 villages in Manujpur. From among those 50 villages, we selected four case study villages in which to carry out longitudinal qualitative research. We chose villages that varied by remoteness and social marginalization: Two villages were midway from town (approximately 8 km) and two were far from town (approximately 16 km); two villages had a typical mix of “other Hindu” (middle or upper caste), SC, ST and Muslim, while two villages were composed predominantly of marginalized groups (SC, ST and Muslim).

We conducted 74 in-depth interviews and 18 focus group discussions (Table 2). Fifteen respondents were interviewed multiple times over the research period to better understand evolving perspectives on the VHSNC and to follow up after specific events. The respondents to whom we returned (13 for a second interview and two for a third) were selected based on their rich prior interviews and experience of VHSNC-related activities. The interviews followed up on the same topics explored throughout the study, described below.

**Table 2:** Interviews and focus groups by respondent type and gender

In-depth interviews	# interviews (# respondents)		
	Male	Female	Total
VHSNC			
• General community	21 (13)	11 (8)	32 (21)
• ASHA	0	8 (6)	8 (6)
• Anganwadi staff	0	12 (9)	12 (9)
• Ward member	3	2	5
Total interviews with VHSNC members	24 (16)	33 (25)	57 (41)
Non-VHSNC			
• General community	4	1	5
• ANM	0	3	3
• ASHA supervisor	1	1	2
• Block Chief Medical Officer	1	0	1
• NGO staff	3 (2)	3	6 (5)
Total interviews with non-VHSNC members	9 (8)	8	17 (16)
Total in-depth interviews	33 (24)	41 (33)	74 (57)
Focus group discussions	# of groups		
	Male	Female	Total
VHSNC (general community, ASHA, AWW, ward)	4	8	12
Non-VHSNC (general community)	3	3	6
Total focus group discussions	7	11	18

We observed 54 VHSNC activities over the course of the intervention, including NGO staff trainings and meetings, village mobilization about the VHSNC, monthly VHSNC meetings, and quarterly cluster level meetings (involving representatives from 17 VHSNCs). Observations were documented to assess participation (e.g. how many people, gender, caste), group dynamics (e.g. who spoke, who was silent, where people sat), and discussion content (e.g. issues discussed, decisions and actions taken).

The interviews and focus groups were conducted in Hindi, primarily by a male Indian researcher who resided in Manujpur for the research period (fifth author). Data collection was closely supported by a female Canadian research coordinator (first author) and a female Indian researcher (fourth author), who also conducted seven interviews and co-facilitated all the focus groups with women. All researchers had master’s degrees in public health or social science subjects and were trained in qualitative research methodology.

Interview and focus group guides asked about: the village context (e.g. gender and caste relations, prior collective action, engagement with government services), VHSNC embeddedness and inclusiveness (e.g. reasons for participation and drop out), VHSNC activities (e.g. experiences in VHSNC meetings and trainings, activities attempted), and public system responsiveness (e.g. how authorities respond to the committee, committee successes or frustrations when seeking change).

Interviews and focus group discussions were audio recorded with the participants’ consent and translated and transcribed into English. All translations were checked and approved by a researcher fluent in Hindi and English and occasionally re-checked during data analysis against the original audio to confirm particularly nuanced or complex passages.

### Data analysis

Initial analysis of the data was guided by thematic network analysis [46] and subsequent interpretation informed by the theoretical concept of social spaces. Thematic network analysis allows researchers to order and synthesize data (e.g. transcripts and observation notes) around thematic variables of interest by first “tagging” portions of text (i.e. sentences or paragraphs) with a short code that indicates the topic, then grouping coded text and synthesizing the content around larger themes. Thematic analysis began with the close reading of the data and development of a coding framework (list of codes with their definitions, grouped by topic). After developing, testing, and refining the coding framework, we applied it to all the transcripts using the qualitative data management software ATLAS.ti. We then read coded outputs to identify higher level organizing themes, such as “female perspectives being heard in the VHSNC”. These organizing themes were developed and substantiated with many example quotations in a descriptive report.

For this paper, to explore how power inequalities were mediated through the VHSNC, the descriptive report was re-examined using the conceptual lens of social spaces. As a theory emerged on how the social space of the VHSNC generated and constrained possibilities for identity and discourse, we re-read the coded outputs, organizing themes, and descriptive report to examine data on gender and notions of responsibility for social services. This re-reading ensured that counter-narratives and alternative explanations were considered and reconciled into the overarching argument of the paper. We identified two central themes, which serve as the headings in the findings section: how participation in the VHSNC renegotiated power relations within the community and how power relations between the community and outside stakeholders were mediated through a discourse of local responsibility.

## Results

### Mediating power relations within the community

As an alternative social space, the VHSNC proposed new physical configurations, particularly by inviting both genders to occupy a public sphere. Bringing together men and women for meetings was largely unprecedented; public meetings to resolve local problems were traditionally for men only. During our research, women explained that attending VHSNC meetings was difficult because of their domestic responsibilities but that they continued to attend nonetheless. Women consistently composed over 50% of meeting attendees, even if most rarely or never spoke. Physical presence is a crude measure of participation [21], but in this context, the VHSNC’s basic gender inclusiveness was a radical variation from established norms. In terms of caste dynamics, powerful VHSNC members, such as men and higher caste people, appeared to accept the involvement of lower-status people. This acceptance partially stemmed from the fact that the VHSNC did not enable access to significant power and money. A higher caste woman said that her VHSNC allowed a marginalized (scheduled caste) woman to participate only because the facilitator insisted and because “it’s not like anyone is getting a salary for this” (Shadeeka, female, other Hindu, IDI_VHC_25). Nonetheless, most respondents strongly endorsed the need to include men and women, as well as representatives from all caste and religious communities.

The transformative potential of women’s opportunity to occupy public space alongside men through the VHSNC was somewhat diminished by the normative justifications given for this policy. Men and women explained that women belonged on the VHSNC because they were responsible for reproductive and child health and men belonged because they could assert demands for improvements and travel outside the village: “Who will listen to the ladies? We need some support from men. The men can only talk with men and other people.” (Shadeeka, women, FGD_COM_03).

While the justifications given for male and female participation in the VHSNC reinforced binary gender norms by valuing women for raising children and men for assertiveness, VHSNC policy and some VHSNC members’ behavior challenged these norms. MoHFW guidelines designated the (female) ASHA as VHSNC secretary and convenor, which demanded capacities unrelated to a domestic or maternal identity: public speaking, leadership, literacy and numeracy, and calling members together for meetings. Many ASHAs struggled with this role, explaining they could not speak to men to call or lead meetings. For example, an ASHA initially said that the VHSNC would not function without support from the NGO facilitator:

Because when he [NGO facilitator] calls, two-or-three men gather. If I go to call people only two-or-three women come, [and] they are illiterate. So in this way it cannot work. Most of them don’t come if I call them (Jhorkibas, female, ASHA, other Hindu, IDI_VHC_29).

However the NGO’s support and training enabled some ASHAs to envision taking a more active role. Six months later, the ASHA quoted above reported that her confidence had grown and that she envisioned herself performing the NGO facilitator’s role:

*Interviewer (male)*: Okay, like you were saying that Rahim [NGO facilitator] does all the talking and writing work. If he doesn’t come who will do it?

*ASHA*: ASHA will do it.

*I*: You will do it! Okay. But see, you don’t talk with men then how will it be possible?

*ASHA*: Then we will do it. I am saying then we will do it. Right now we can see that he is doing it. When we have to, we will do it. We will have to do the meeting and we will have to raise the issues. (Jhorkibas, female, ASHA, other Hindu, IDI_VHC_48)

In addition, female VHSNC members began taking action outside the VHSNC on issues beyond reproductive and child health. In particular, following VHSNC training, some female VHSNC members worked to improve the village schools, which were staffed by male teachers from outside the community. In one village, female VHSNC members asserted the right for local girls to be admitted to the secondary school through a government scholarship program. In another village, a female VHSNC member demanded that the headmaster release government grant money for schoolgirls to buy bicycles:

VHSNC member Rashmi’s daughter had not received money for her bicycle. Rashmi followed up with the headmaster several times so eventually he said to Rashmi, ‘Tell me your daughter’s name and take her cheque but don’t disclose this to anybody.’ Rashmi said to headmaster, ‘I have 22 daughters studying here in this school. So you have to give their respective money to all.’ After three days, the headmaster gave the bicycle money to all the girls. (Observation of VHSNC cluster meeting in Sojjanpur, respondent from neighboring village, OBS_VHC_15)

Another female VHSNC member in Sojjanpur village discussed women’s capacity to challenge the teachers for “lazing around” and “playing cards” in the day. She explained:

It is because of these meetings that we can move forward… Otherwise we can’t even climb up to the gates of the school… So teachers also get to know sometimes that if any ladies come, they feel we can also be in control. Because of this we participate. (Sojjanpur, female, Muslim, IDI_VHC_38)

Male VHSNC members tended to take action within accepted masculine spheres, primarily by raising requests to government agents for a range of issues (particularly access to potable water and improved health services). But several men also took interest in monitoring anganwadi preschool services, a traditionally feminine domain. Male VHSNC members reported that they checked the center to see if food was being provided to children. However, their monitoring was solely visual, since they could not converse with the (female) anganwadi staff.

NGO staff made efforts to actively include women’s voices during VHSNC meetings and trainings. During these events, female members sat clustered together silently or whispering among themselves. NGO staff encouraged and cajoled the women to present their views. By suggesting that female participation in front of men was normal and expected, the VHSNC space could be a site of renegotiating gender norms for greater female influence. It could also enable women who wanted to speak to do so, under the guise of having been forced by the NGO facilitators. However, the NGO staff’s efforts to make women speak also pushed women to violate norms, which could have negative consequences for women upon leaving the “alternative space” of the VHSNC.

Women managed this risk in several ways. Sometimes, they offered quiet one-word replies to deflect attention without overtly ignoring the NGO facilitator’s request. But often the group of women (including the ASHA) discussed issues amongst themselves and then the ASHA presented their collective opinion. Male VHSNC members appeared to accept this micro-violation of gender norms, perhaps because men recognized that the NGO facilitator had forced the point, rather than the women themselves exhibiting boldness and a desire to be heard, which would be unacceptable. In addition, it was accepted as appropriate for ASHAs to occasionally speak in front of men to maintain their socially valued (government, salaried) work.

We also witnessed occasional “slippages” in gender performance [47], when (non-ASHA) female VHSNC members interjected in meetings. For example, one female VHSNC member recalled how a woman in labor delivered a stillborn baby while waiting for an ambulance (Sojjanpur, OBS_VHC_29). As the possibilities of gender are “necessarily constrained by available historical conventions,” [47], these instances where female participants failed to re-enact expected gender behavior pressed the boundaries of acceptable performance.

Challenging gender norms within the VHSNC did not protect women from experiencing consequences when they returned to everyday life. Informal social processes, enacted by both men and women, police gender performance to maintain the *status quo*. An NGO facilitator explained that in one village, women were scolded at home for speaking up and were no longer allowed to participate:

The Muslim women were coming for the meetings and at times they even went for training… [But] if they put forward some viewpoint of their own, at times it so happens that they get scolding back home for saying such things… They [family members at home] said ‘now no one will want to marry the girls in our family. We will have a bad name in the village.’ Now the women are not allowed to come for any meetings (female, other Hindu, IDI_OTHER_05).

Another facilitator (female, other Hindu, IDI_OTHER_06) said that women were afraid to speak “because they think that after meeting the men can say that you were speaking too much.” In one instance, men expressed their discomfort with subtle challenges to gender relationships within the VHSNC. When NGO staff made an additional effort to encourage women to attend trainings, men laughed at the prospect of women taking leadership roles, seeking to reinforce the absurdity of the concept: “Women will take the training, will work as officers in the committee and we will be their peons. [All laughing]” (Hanwari, men, FGD_VHC_06).

### Mediating power relations between the community and outside actors

Power relations between the community and outside actors manifested most starkly in the construction of a “discourse of responsibility” for improving health, sanitation, and nutrition in the villages. Powerful outside stakeholders (MoHFW policymakers, NGO staff, health system functionaries) sought to present the VHSNC as a viable participatory body by framing VHSNC members as actors responsible for and capable of effecting local change.

The MoHFW VHSNC guidelines suggested that the VHSNC focus village level health action, recommending that members “gather and clean the village” and “organize teams for source reduction work” to stop mosquito breeding in stagnant water [30]. It also positioned the VHSNC as capable of acting to improve local health through “informing local authorities” so that “health care delivery and public services are improved upon” (*ibid*). Health system functionaries repeatedly emphasized local responsibility. For example, the Block Chief Medical Officer (BCMO) told VHSNC members that they were responsible for overseeing the auxiliary nurse midwife (ANM):

*BCMO*: At the sub center you have the responsibility to ask the ANM where she is working, the status of medicines, and the care given to pregnant women and children. It is also the VHSNC members’ responsibility to be aware of the services available at your sub center. For example sub center should be open from 9 AM to 11 AM and in this time period sick people can go to the center and gain health services. (Observation note, VHSNC cluster meeting, OBS_VHC_24)

NGO staff suggested the VHSNC could take responsibility for checking the functionality of the Manujpur hospital and for having health worker vacancies filled:

*SEEK director (female):* Here decentralization has the advantage that people or committee members have the authority to monitor these local institutions. For example, in the Manujpur CHC [community health centre], the government is providing Rs. 30,000 [US $550] for cleanliness. But can you see the outcome of that money? That CHC is always dirty. Here it would be your responsibility to check that the hospital is functioning the way it should be.

*SEEK field manager (male):* There are many problems in the health sub-centers, such as whether an ANM is appointed or not. If an ANM is not appointed, it the responsibility of the committee is to write a proposal to the government for ANM appointment. (Observation note, VHSNC cluster meeting, OBS_VHC_31)

Most VHSNC meetings, led by the NGO facilitators, focused on identifying service gaps and writing requests to the authorities to address these gaps. Whenever VHSNC members were able to speak to government agents, members requested service improvements, such as health workers, medicine, and equipment at health centers, and improved water, drainage, roads, and waste management. Overwhelmingly the government response focused responsibility back on the VHSNC by telling members to write additional requests to higher level government agents, to follow up with various departments, or to solve the problem themselves.

Many VHSNC members felt that the discourse of local responsibility resonated with their worldview, but this agreement came with adverse consequences for collective agency. Those who accepted local responsibility for improving village health, sanitation, and nutrition had to find ways to explain why so few improvements occurred. They did so by blaming their peers for lacking the positive attributes necessary to fulfill this responsibility.

Many agreed that if only the village had sufficient “social feeling” (male, ST Hindu, Hanwari, IDI_VHC_54), then a great amount could be achieved through the VHSNC. A male VHSNC member blamed the community’s “lack of initiative” for the fact that the untied fund was never released for their use, although the VHSNC wrote numerous requests and asked a number of government agents about the money:

In the meeting we were informed about the fund, but the members do not take initiative or responsibility to know whether the money has been transferred or not, and how to use the money. (Sojjanpur, male, SC, IDI_COM_06)

Respondents said people were “stingy,” only willing to work for personal gain, and uninterested in the VHSNC when they “realized that they would not get anything” (Jhorkibas, ASHA, other Hindu, IDI_VHC_29), despite examples of families working together to help people reach the hospital or access water. A male member blamed illiterate women who “cannot understand things” for throwing their garbage in public spaces of the village, despite later noting that there was no alternative waste management system in place (Sojjanpur, male, SC Hindu, IDI_VHC_45). Overall, he felt that the “progressive” and “literate” people in the village were up against a majority of “illiterate boors” who impeded VHSNC efforts to better the village.

Accepting the dominant discourse of local responsibility thus had a high social cost. If the VHSNC was truly responsible for improving the village, the only way to explain poor outcomes was to blame one’s community for failing to take up this responsibility and improve the village. This perceived failure then reinforced a sense that nothing could improve because of the community’s collective failings.

A sizable minority of VHSNC members, particularly male members who stopped participating, resisted dominant claims of local responsibility. These (largely inactive) VHSNC members provided numerous examples of upstream problems that VHSNCs lacked the capacity to solve. They explained that the VHSNC brought no new political power for local people to demand change from the government and no new financial capacity for local people to fix problems themselves. They rejected the idea that any village problem could be solved by village residents taking responsibility, noting that improved water access required expensive development beyond the village’s means, that stagnant water gathered because of poor drainage infrastructure, and that improved waste management required the public works department to clear open air garbage piles.

For instance, in a focus group discussion with men who refused to attend VHSNC meetings, the group derided the notion of lending their time and energy to direct civic maintenance, asking: “What to do on sanitation? Shall we take brooms individually and make the village clean or what?” (Hanwari, male, FGD_VHC_06). In another instance, a VHSNC member explained that they had exhausted the avenues available to them to fill their village’s vacant ANM position, and suggested that responsibility lay with politicians:

*Male VHSNC member*: Nothing is going to happen. We have been waiting for six years in our village, but ANM recruitment still has not been done. The CMO [Chief Medical Officer] clearly said recruitment will only be done with the help of politicians. So whom should we consult with? We don’t have money to go to [state capital] or Delhi. (Observation note, VHSNC meeting, Shadeeka, OBS_VHC_24)

Another group of male VHSNC members explained that they needed government support, but pointed out that the administration avoids responsibility by telling villagers that the community must take up the work: “the administration gets away with inaction by saying that this is your work” (Sojjanpur, male, FGD_VHC_12). The men spoke at length about the need for greater government engagement in the VHC:

*M3*: The truth is one person cannot do anything. Our committee cannot do anything. The village is also with us but until the department is with us nothing can be done.

*M1*: True

*M3*: If the department is with the committee, then there will be a solution. But neither PHED [Public Health Engineering Department] is with us, nor the PWD [Public Works Department] nor the Health Department is with us. (Sojjanpur, men, FGD_VHC_12)

## Discussion

**Fig. 2:**
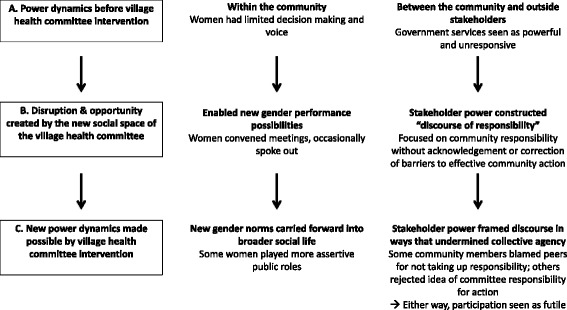
Renegotiation or reinforcement of power relations through village health committees

In this paper, we examine health committees as initiatives where power relations within communities and between communities and outsiders may be renegotiated or reinforced, focusing particularly on gender and discourses of responsibility (Fig. 2).

In terms of power relations within communities, we identified a number of opportunities that enabled subtle challenges to pre-existing restrictive gender norms. First, requiring a mixed-gender group and expecting them to communicate about valued services (health, nutrition, and sanitation) was a radical act in a community without any other forums for cross-gender collective dialogue and decision making, even if most women did not verbally participate. Second, some members used their identity as VHSNC members to push the boundaries of acceptable feminine or masculine concerns and behaviors: as VHSNC conveners, ASHAs took up leadership positions, and several female VHSNC members publically asserted themselves to improve girls’ access to education. The VHSNC also made it possible for male members to enter the traditionally female space of the anganwadi center and take interest in the quality of food and care provided there. Third, when some women exercised voice in the VHSNC, it contributed to normalizing the idea of women speaking in the presence of male community members.

It must be noted however that although the VHSNC opened up some new possibilities and enabled some women to express their own and collective female voices, for most women speaking openly in front of men continued to be unacceptable. Many female participants remained silent, cognizant of the everyday reality awaiting them outside the VHSNC, and spoke only with their physical presence. Women who pushed the boundaries of acceptable behavior—at times invited and cajoled into doing so by NGO facilitators—could not be protected from conflict upon returning home, where dominant interests were invested in sustaining the *status quo*. The transformative potential of VHSNCs to serve as “alternative spaces” where new identities are rehearsed was constrained by the limited time in which participants entered the space and the participants’ awareness that they had to return to their everyday power relations as soon as the meetings ended [35, 48]. Nonetheless, social change can be a series of micro-transgressions, each of which presses the boundaries of acceptable performance and all of which add up to alternative norms. The VHSNC created such opportunities for change as female, and occasionally male, VHSNC members re-interpreted their social roles.

In terms of power inequalities between communities and outside stakeholders, the discourse of local responsibility played out in VHSNCs as both an instrument of power used by outside stakeholders and as a point of resistance.

Participants who accepted the discourse of local responsibility reconciled it with the lack of local action by suggesting that their peers were selfish and unwilling to take collective action. The VHSNC thus re-entrenched disempowering collective identities. Our findings resonate with experiences in Tanzania, where participants on community malaria control boards became increasingly disparaging of their peers [49]. Board members adopted the language of outside elites who explained the poor health of marginalized people through their incapacity to solve their own problems (*ibid*). In this sense, VHSNCs can be “invited” spaces [22], created and defined by dominant actors to generate agreement around pre-determined agendas and further a dominant discourse [27].

However, power is a disparate and creative force abounding with resistance [37] and community members can resist the agendas of participatory development programs [39]. In our study, some community members contested the dominant justification for the VHSNC by presenting an alternative discourse of upstream responsibility. We note that this resistance, no matter how compelling and justified, overlooked the fact that some VHSNCs did take local actions to support or monitor the anganwadi worker and schools, and to affect change, or to coordinate political efforts to push for improved services. There is a need for renegotiating the discourse around VHSNCs to create space for communities’ legitimate anger at poor services and desperate need for improved upstream support, without closing the possibility for smaller scale local action within the community. Whether accepting or rejecting the discourse that local people can improve their health, sanitation, and nutrition through the VHSNC, most people planned to stop participating.

There are two central implications of these findings. First, participatory initiatives such as the VHSNC can create opportunities to challenge power inequalities within communities, even in contexts with rigid gender norms such as Manujpur. This finding resonates with research linking women’s participation in social groups to improved health and development outcomes [50] and the creation of space for critical dialogue on gender leading to more equitable behavior in the real world [51, 52]. Facilitation by the outside NGO staff emerged as essential to constructing VHSNCs as alternative social spaces where new rules and possibilities can be ascribed. This finding is a valuable affirmation of the transformative potential of participatory programs, in light of concern that they are prone to elite capture and the exclusion of marginalized people within villages, and suggests that policymakers should prioritize ongoing high quality facilitation. The strategies used in other interventions to generate transformative social spaces for collective dialogue and critical reflection [35, 36, 53] can inform future VHSNC policy and programming. However, we must not overstate the potential of micro-disruptions to power relations. Women and lower caste people continue to face major barriers to full participation and self-determination in the decisions that affect their lives.

Second, greater attention must be paid to the fundamental link between participation in VHSNC activities and genuine empowerment through gaining control over the resources needed for increased opportunities. Campbell [40] highlights that decades of community mobilization have focused on empowerment to overcome symbolic aspects of oppression (such as negative self-narratives) without adequate attention to the materialist roots of oppression (i.e. economic inequality). Just participating in meetings and discussions of health, sanitation, and nutrition issues is of little value without the tools to address these issues.

VHSNC policy thus needs to genuinely empower VHSNCs, for example by ensuring access to meaningful funding and improving health system responsiveness to VHSNC demands. However, as VHSNCs gain greater power, the stakes associated with VHSNC participation may rise. More powerful members may try to edge women and lower caste people out and be less tolerant of micro-violations of norms. Ongoing support through skilled facilitation and research to assess positive and negative consequences as VHSNCs gain increasing power will be vital to sustain gains and mitigate risks.

## Conclusion

This study examined VHSNCs as social spaces where power relations are negotiated, to understand their transformative potential. We found that with support from NGO facilitators, VHSNCs enabled members to try out new gender roles, which to a small extent carried forward into everyday life. However, the rationale for participation was set by powerful outside stakeholders, who emphasized community responsibility for improving health without acknowledging or changing structural and practical barriers beyond the community. This “discourse of responsibility” was accepted by some community members and resisted by others, but either way reinforced a negative collective identity, thus hindering the VHSNC’s transformative potential.

Power is *always* at play in social relations and thus must be thoughtfully utilized to promote social justice, including in participatory initiatives [35]. For VHSNCs to support social transformation, they must serve as social spaces where participants can practice more equitable within-community power relations and where power inequalities between communities and outside actors are challenged through legitimizing collective community experiences of marginalization and engaging powerful outside actors to reduce material inequities. Great potential to extend the VHSNC’s transformative potential lies in sustaining the gender and caste co-occupation of space and continuing to build women’s voice, while at the same time strengthening the committee’s economic and political power. However, challenging power relations is not without risk and (often unintended) consequences. Ongoing monitoring, advocacy, and supportive facilitation are critical to ensure that VHSNCs achieve their transformative potential.

## Abbreviations

ANM: Auxiliary nurse midwife
ASHA: Accredited social health activist
BCMO: Block chief medical officer
CHC: Community health centre
FGD: Focus group discussion
IDI: In-depth interview
MoHFW: Ministry of Health and Family Welfare
NGO: Non-governmental organization
SC: Scheduled caste
ST: Scheduled tribe
VHSNC: Village health, sanitation, and nutrition committee

## Footnotes

1 We are using pseudonyms for the study area, villages, implementing NGO, and all respondents to protect anonymity
